# Development of Stereotypic Behaviors and Personality Traits of Captive Male Forest Musk Deer and Relationships with Musk Secretion

**DOI:** 10.3390/vetsci13030261

**Published:** 2026-03-11

**Authors:** Xiaoping Lu, Yan Sheng, Hong Ye, Zisong Yang, Xiuxiang Meng

**Affiliations:** 1 School of Ecology & Environment, Renmin University of China, Beijing 100872, China; 2 School of Resource & Environment, Aba Teachers College, Wenchuan 623002, China; yangzisong@126.com

**Keywords:** forest musk deer (*Moschus berezovskii*), *ex situ* conservation, muscone, stereotypic behaviors, exploration level, activity level

## Abstract

Forest musk deer (*Moschus berezovskii*) are endangered ungulates, and captive breeding has been effective for its *ex situ* conservation and sustainable medicinal musk utilization in traditional Asian traditional medicine. Methods of focal sampling and all-occurrence recording were used to evaluate the intensity of stereotypic behaviors and personality traits in 25 captive male forest musk deer. Additionally, musk secretion was monitored in this study, and muscone content was measured with gas chromatography, thereby analyzing the interrelationship between their stereotypic behavior, personality traits, and musk secretion. The results revealed no significant differences in stereotypic behaviors or personality traits across age groups (*p* > 0.05). Individuals with stereotypic behaviors showed a tendency toward secreting higher amounts of musk and muscone content compared to those without stereotypic behaviors, although these differences were not statistically significant (*p* > 0.05). Furthermore, the exploratory behavior level and active behavior level were not significantly correlated with the secretion amount of musk. However, both the exploratory behavior level and active behavior level exhibited significant positive correlations with muscone content (*p* < 0.05). These findings provide valuable insights for the breeding, welfare, and *ex situ* conservation of endangered forest musk deer.

## Simple Summary

Musk deer are shy and easily stressed, and thus struggle to adapt to artificial environments compared to other captive animals. This stress can lead to them performing unnatural, repetitive behaviors. Understanding their individual personalities could help us improve their welfare and manage them better. In this study, we watched the behavior of 25 male forest musk deer living on a farm in China. We recorded how often they performed repetitive actions and assessed their personalities, categorizing them as more exploratory and active or more cautious. We also measured the amount and quality of the musk secreted by the male musk deer, a valuable substance used in medicine. We found that musk deer with more exploratory and active personalities produced higher quality musk. The repetitive behaviors were generally mild and did not reduce their musk production. This suggests that an animal’s personality could serve as a useful guide for breeding programs. More curious and active individuals might be better suited for captive farms, while shyer individuals might be more appropriate candidates for release into the wild. These findings offer a simple way to help conservation efforts for this endangered species.

## 1. Introduction

Musk deer (*Moschus* spp.), a group of small solitary forest ruminants (SSFRs), are endangered resource species endemic to Asia [1,2]. The musk secreted by male individuals is widely utilized in traditional Asian medicine and the perfume industry, rendering it a threatened resource species [3]. Due to historical overexploitation and habitat changes, wild populations of musk deer have become critically endangered [2]. Musk deer species are listed on the Red List of the International Union for Conservation of Nature [4] and in the Appendix of the Convention on International Trade in Endangered Species of Wild Fauna and Flora (CITES) [1].

*In situ* strategies (e.g., habitat protection) have been insufficient due to ongoing poaching and habitat loss [5]. *Ex situ* management, particularly captive breeding, has been crucial for musk deer conservation and the sustainable use of musk [6,7]. China’s captive breeding programs have currently produced over 60,000 individuals and enabled reintroduction efforts [5,8]. The forest musk deer is the most numerous *Moschus* species in captivity, followed by the alpine musk deer (*Moschus chrysogaster*) [7]. The healthy development and sustainable production of captive musk deer populations have received widespread attention [8,9]. The musk production capacity can be reflected by indicators such as the secretion amount of musk and muscone content [10].

To enhance the scope of *ex situ* conservation and scale of musk production, forest musk deer have been introduced to Fujian Province in southeastern China, in addition to breeding efforts being undertaken in their natural habitats, such as in Sichuan and Shaanxi Provinces [11]. This initiative has contributed to the establishment of the easternmost *ex situ* conservation population of forest musk deer [7,12]. In male forest musk deer, musk secretion typically begins around 1.5 years of age and gradually increases during subsequent years, reaching relatively stable levels by 3.5 years [10]. Therefore, the 1.5–3.5-year age range represents a critical period from initial musk production to early maturity, making it suitable for examining the relationships between behaviors, personalities, and musk secretion [13]. Understanding stereotypic behaviors and personality traits is important because they affect animal welfare, reproductive success, and musk production in captivity [14]. Improving welfare and productivity supports sustainable *ex situ* populations, which in turn can provide individuals for reintroduction into the wild. However, there is a lack of behavioral and physiological research concerning captive forest musk deer in the eastern regions of China, particularly regarding the relationship between their stereotypic behaviors, personality traits, and musk secretion [12,13].

The environment in which captive animals are kept is inherently artificial, characterized by limited space and scheduled feeding, which can easily lead to the development of stereotypic behaviors [15,16,17]. Studies have shown that the manifestation of these behaviors can adversely impact both animal welfare and the productivity of captive animals (such as with regard to meat, eggs, milk production, etc.) [18]. However, some studies have shown that this correlation does not always exist [19]. For instance, cows (*Bos taurus*) exhibiting stereotypic behaviors may yield more milk than normal cows, as their activated energy metabolism mobilizes body nutrient reserves to support lactation, while their more stable lying and drinking behaviors and stronger heat tolerance collectively enhance lactation performance [20]. A study on captive alpine musk deer has also found no significant correlation between stereotypic behavior and musk secretion [14].

Furthermore, personality traits can represent stable behavioral trends in animals across time and situations among individuals, and are closely related to resource use, reproduction, and predation [21,22,23]. For example, male zebrafish (*Danio rerio*) with heightened levels of boldness and aggression tend to possess a competitive advantage during the breeding process [24]. Some studies have analyzed the personality of musk deer but have not associated it with musk secretion [25,26]. Another study focused on identifying boldness–shyness traits in forest musk deer using behavioral tests, but did not examine how these traits relate to musk production [25]. Moreover, one study explored the link between boldness and hormone levels in alpine musk deer, but did not extend their analysis to musk secretion [26]. Thus, although these studies advanced our understanding of personality in musk deer, they left a gap in evaluating whether personality traits influence musk yield or quality—an important consideration for integrating behavioral indicators into breeding and management strategies.

This study, based on the behavioral sampling of individuals under human care with the lowest latitude, investigated the stereotypic behaviors and personality traits of forest musk deer and their relationships with musk secretion. This study aims to (1) quantify the types and intensities of stereotypic behaviors in the captive male forest musk deer population; (2) assess the levels of personality traits, including exploration level, activity level, self-directed level, and redundancy level; (3) analyze the differences in the above indicators among different age groups; and (4) explore the relationships between the stereotypic behaviors and personality traits with the musk secretion of forest musk deer. And we hypothesized the following: (1) Stereotypic behavior intensity and personality traits differ among age groups. (2) There are differences in the musk secretion (amount of musk, muscone content) among individuals with different personality traits, e.g., individuals with a higher exploration level produce more musk and higher muscone content. (3) Stereotypic behaviors are associated with reduced musk secretion. The research findings can provide important references for the *ex situ* conservation, breeding, and musk production of this threatened species.

## 2. Materials and Methods

### 2.1. Research Area

This study was conducted from 1 September to 20 December 2023 at a musk deer farm located in Zherong County, Fujian Province, China. The musk deer farm is situated in the southeastern inland mountainous area of Fujian Province (27°05′ N~27°19′ N, 119°43′ E~120°04′ E), with an average altitude of about 850 m, an annual average temperature of 16 °C, and an annual average rainfall of 2100 mm. This population represents the easternmost and lowest-latitude captive forest musk deer population in China, established through introduction from traditional breeding areas in Sichuan and Shaanxi Provinces.

### 2.2. Research Population

This study involved 25 male captive forest musk deer, with an age range of 1.5–3.5 years. This age range was based on the actual breeding situation of the newly established musk deer farm, where the introduced individuals had a relatively young age, and no adult individuals over 3.5 years old were available at the time of the study. Notably, all individuals had reached the musk-secreting stage, which ensured the uniformity of the core research index (musk secretion) and the validity of the subsequent behavioral and physiological correlation analysis. The individuals were further divided into three age groups with clear sample sizes: 1.5-year-olds (*N* = 8), 2.5-year-olds (*N* = 10), and 3.5-year-olds (*N* = 7).

Each musk deer was kept alone in a 10 m^2^ enclosure and a 30 m^2^ activity area. Their health condition was good, and ear tags were used for individual recognition. The feed composition comprised concentrate (40% corn, 12% wheat bran, 25% soybeans, 15% soybean meal, and 8% whey powder) and fresh green leaves (such as leaves of *Morus alba* and *Taraxacum mongolicum*). The feed was provided twice daily (at 05:00 and 17:00), and the enclosures were cleaned during feeding, with no disturbances to the musk deer at other times.

### 2.3. Behavior Sampling

Following reference [1], focal sampling and all-occurrences recording were employed to record individual behavior categories and durations. Observations were carried out outside the enclosures using binoculars (10 × 50) to minimize disturbance, as recommended for behavioral studies on captive ungulates [9]. Each focal sampling session lasted 10 min per individual, conducted during the peak activity periods of forest musk deer (05:00–08:00 and 17:00–20:00). A total of 25 individuals were observed, and each individual was sampled repeatedly across different days throughout the study period to obtain representative behavioral data. If a focal animal moved out of sight during a session, sampling was paused and later rescheduled to ensure complete records [27].

Stereotypic behaviors observed in this population were defined based on the established ethogram for captive musk deer [9] (Table 1). The stereotypic behaviors can be categorized into oral stereotypic behaviors (e.g., eating non-food items and stereotyped licking and scraping) and locomotor stereotypic behaviors (e.g., galloping, to-fro-walking, platform standing, wall jumping, and stereotypic gazing).

The exploration level of personality traits is reflected by the duration of forest musk deers’ exploratory behaviors, which include environmental sniffing and anogenital sniffing. The activity level is reflected by the duration of non-resting behaviors, which include moving (stepping, walking, trotting, running). The self-directed level is reflected by the duration of self-directed behaviors, which include self-grooming and self-scratching [9].

The duration proportion of stereotypic behaviors (SBs) was used to evaluate the stereotypic behaviors intensity (SBI) [9]. Similarly, the duration proportions of exploratory behaviors (EB), active behaviors (AB), and self-directed behaviors (SDB) were used to evaluate personality traits: exploration level (EL), activity level (AL), and self-directed level (SL), respectively [28]. The behavioral redundancy level (RL) was calculated as the complement of the behavioral diversity index (BDI) [29]. Thus, the stereotypic behavior intensity (SBI) and four personality traits (EL, AL, SL, RL) are calculated in this section. The formulas are as follows: $SBI=\frac{Duration\;of\;SB}{600\;s},\;EL=\frac{Duration\;of\;EB}{600\;s},\;AL=\frac{Duration\;of\;AB}{600\;s},\;SL=\frac{Duration\;of\;SDB}{600\;s}$, and RL=1−BDI.

### 2.4. Musk Collection and Muscone Measurement

A process table was drafted before musk collection, and the necessary tools were prepared, such as a sterile spoon and tray, the electronic scale, and sealable sampling bottles. After each musk deer was manually restrained by experienced keepers, the spoon was used to gently probe into the musk sac and extract the musk via rotation. The duration of the procedure was approximately 2–3 min per animal, and no distress could be observed, only mild struggling and an increased heart rate; this has been proven to be a non-invasive and sustainable harvesting procedure [30,31]. Then, the collected musk was placed on a tray for weighing and then stored in a sealed bag. The secretion amount of musk (SAM) collected from an individual musk deer was accurately recorded.

The muscone content (MC) was determined using the gas chromatography method [32]. A Shimadzu Nexis GC-2030 gas chromatograph (Shimadzu Corporation, Kyoto, Japan) equipped with an SH-Rtx-5 capillary column (30 m × 0.25 mm × 0.25 μm) was used. The test solution was prepared as follows: Take approximately 0.05 g of the dried sample, weigh it accurately, add 2 mL of anhydrous ethanol, seal it, and sonicate it for half an hour. Let it stand for 1 h, and then filter it to obtain the solution. The reference solution was prepared as follows: Take an appropriate amount of muscone reference substance, weigh it accurately, and dissolve it in anhydrous ethanol to prepare a solution of 1.5 mg·mL^−1^. The injection volume is 1.0 μL, and the solvent and sample are rinsed once and twice, respectively, before injection [33]. The temperature is set at 25 °C, the carrier gas is nitrogen, the pressure is 114.0 kPa, the total flow rate is 50.0 mL·min^−1^, the column flow rate is 1.91 mL·min^−1^, the equilibrium time is 3.0 min, and the tailing flow rate is 24 mL·min^−1^.

### 2.5. Statistical Analysis

The Shapiro–Wilk test was used to assess the normality of the data for all measured variables: stereotypic behavior intensity (SBI), exploration level (EL), activity level (AL), self-directed level (SL), behavioral redundancy level (RL), secretion amount of musk (SAM), and muscone content (MC). For comparisons among the three age groups (1.5, 2.5, and 3.5 years), one-way ANOVA was used for normally distributed variables (AL, SL, RL), while the Kruskal–Wallis test was applied to non-normally distributed variables (SBI, EL, SAM, MC). For pairwise comparisons between stereotypic and non-stereotypic groups within each age category, independent *t*-tests (for normally distributed data) or Mann–Whitney U tests (for non-normally distributed data) were used.

Pearson’s correlation analysis was used to evaluate correlations among normally distributed variables (AL, SL, RL), while Spearman’s correlation analysis was applied to non-normally distributed variables (EL, SBI, SAM, MC), based on the Shapiro–Wilk test results. The significance level was set at *p* = 0.05, and all data analyses were conducted with SPSS 26.0 [13].

## 3. Results

### 3.1. Stereotypic Behaviors of Captive Forest Musk Deer

Behavioral sampling results indicate that the forest musk deer population in this study developed stereotypic behaviors such as stereotyped licking and scraping, to-fro-walking, platform standing, wall jumping, and stereotypic gazing, and each type is described in Table 1.

### 3.2. Intensities of Stereotypic Behaviors

**Figure 1:**
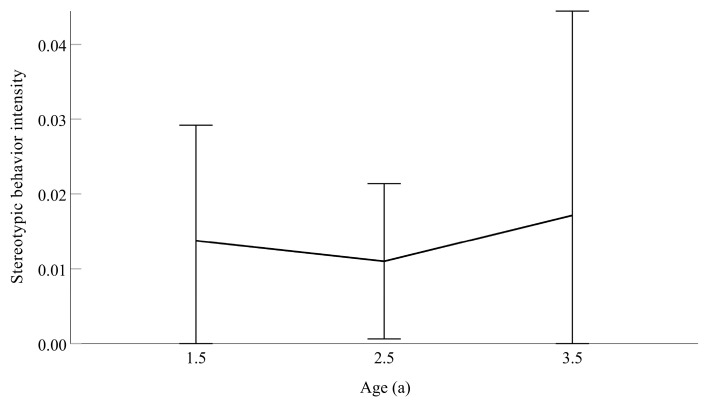
Stereotypic behavior intensity of musk deer in different age groups (1.5-year-olds (*N* = 8), 2.5-year-olds (*N* = 10), and 3.5-year-olds (*N* = 7), respectively).

Analysis of stereotypic behaviors revealed that there is no statistically significant difference in the stereotypic behavior intensity across the three age groups (1.5, 2.5, and 3.5 years) of forest musk deer (*p* > 0.05) (Figure 1).

### 3.3. The Relationship Between Stereotypic Behaviors and Musk Secretion

Not all individuals displayed stereotypic behaviors. The results show that the SAM in the stereotypic behavior group (16.47 ± 6.12 g, *N* = 11) tended to be higher than that in the non-stereotypic behavior group (12.14 ± 4.96 g, *N* = 14), but the difference did not reach statistical significance (*p* > 0.05). Similarly, no significant differences were found in the muscone content between individuals that exhibited stereotypic behaviors (1.75 ± 0.88%, *N* = 11) and those that did not (1.56 ± 1.02%, *N* = 14) (*p* > 0.05) (Table 2).

To further explore the age-specific distribution of stereotypic behaviors, we stratified the samples by age groups and quantified the number of individuals with and without stereotypic behaviors in each group (Table 2). Statistical analysis revealed no significant differences in musk secretion between the stereotypic and non-stereotypic subgroups within each age group (all *p* > 0.05).

### 3.4. Personality Traits of Captive Forest Musk Deer

The correlation among the four personality traits is shown in Table 3. A significant positive correlation exists between EL and AL (Spearman’s *ρ* = 0.780, *p* < 0.05), while the other correlations do not reach significance (*p* > 0.05). In the analysis of the relationship between SB and the four personality traits, SBI exhibited a significant negative correlation with RL (Pearson’s *r* = −0.399, *p* < 0.05), while other correlations remained non-significant (*p* > 0.05).

**Figure 2:**
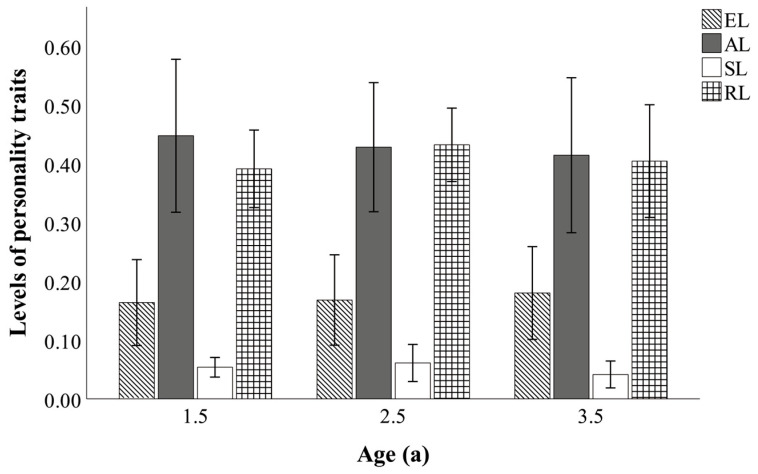
Personality traits of different age groups (1.5-year-olds (*N* = 8), 2.5-year-olds (*N* = 10), and 3.5-year-olds (*N* = 7)). EL, exploration level; AL, activity level; SL, self-directed level; RL, behavioral redundancy level.

The personality traits across various age groups, ranging from 1.5 to 3.5 years, are shown in Figure 2. No significant differences were found among age groups in exploration level, activity level, self-directed level, or behavioral redundancy level (all *p* > 0.05).

### 3.5. Relationship Between Personality Traits and Musk Secretion

Analysis of the relationship between personality traits and musk secretion revealed that EL (Spearman’s *ρ* = 0.262, *p* > 0.05), AL (Pearson’s *r* = 0.128, *p* > 0.05), and SL (Pearson’s *r* = 0.224, *p* > 0.05) showed positive but non-significant correlations with SAM. Similarly, RL demonstrated a negative but non-significant correlation with the SAM (Pearson’s *r* = −0.016, *p* > 0.05) (Table 4).

**Figure 3:**
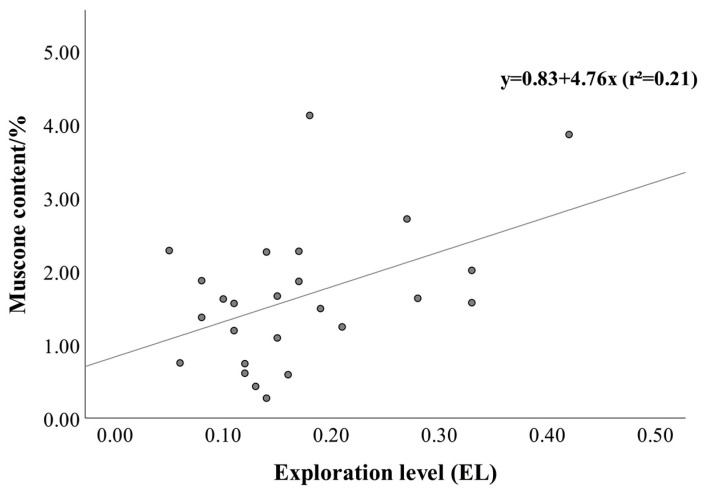
The relationship between exploration level and muscone content.

**Figure 4:**
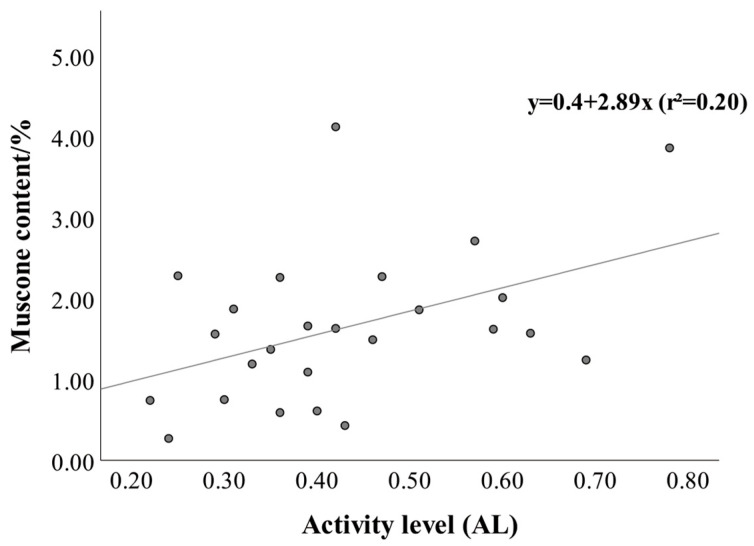
The relationship between active behavior level and muscone content.

In the context of muscone content, EL was found to have a significant positive correlation (Spearman’s *ρ* = 0.461, *p* < 0.05), which can be expressed through the regression equation y = 0.83 + 4.76x (*r*^2^ = 0.21) (Figure 3). Similarly, AL exhibited a significant positive correlation with MC (Pearson’s *r* = 0.443, *p* < 0.05), represented by the regression equation y = 0.4 + 2.89x (r^2^ = 0.20) (Figure 4). Conversely, SL (Pearson’s *r* = −0.288, *p* > 0.05) and RL (Pearson’s *r* = −0.104, *p* > 0.05) were associated with negative but non-significant correlations with MC (Table 4).

## 4. Discussion

Captive environments designed for *ex situ* conservation often restrict natural ranging and foraging, predisposing small ungulates to stereotypic behaviors [34]. This study found that captive forest musk deer in Fujian Province displayed a higher frequency of head stereotypic behaviors, such as stereotypic rubbing. Notably, such head stereotypic behaviors have not been reported in previous studies of captive alpine musk deer, which primarily reported oral stereotypic behaviors (such as eating non-food items, stereotypic licking and scraping) [35]. Given that forest musk deer are smaller in size compared to alpine musk deer, it is speculated that they are more inclined to regulate energy metabolism and release stress through head movements under captive conditions, while alpine musk deer have evolved more oral stereotypic behaviors in restricted activities. This difference may be attributed to species-specific coping styles: smaller-bodied forest musk deer may rely more on locomotor and head movements to dissipate stress, whereas larger alpine musk deer may exhibit more oral stereotypies as a form of displacement activity [36].

In this study, although individuals exhibiting stereotypic behaviors showed a trend of higher secretion amount of musk and muscone content, this difference was not significant. Compared to in previous studies on captive forest musk deer [12,37], the average musk secretion in our study is within the normal range, indicating that the population is not impaired in musk production. This contrasts with some previous research conclusions [37,38] suggesting that the higher intensity of stereotypic behaviors developed in forest musk deer may reduce the behavioral diversity, thereby affecting animal welfare and musk production. In this study, the overall expression intensity of stereotypic behaviors in forest musk deer was relatively low, and the diversity index of individuals exhibiting stereotypic behaviors showed little difference compared to those not exhibiting such behaviors, thus not causing negative impacts on production efficiency.

Some studies have also indicated that, in some instances, low-intensity stereotypic behaviors may have a positive impact on animal welfare and productivity, serving as a form of stress regulation similar to self-enrichment or meditation [18]. Additionally, low-intensity stereotypic behaviors may be a coping mechanism that redirects metabolic resources and energy to key physiological functions, such as musk synthesis [20,39]. This redirection may be mediated by neuroendocrine changes, such as altered cortisol levels, which prioritize energy allocation towards reproduction or secretion [40]. For example, in this musk deer farm, individuals with relatively high stereotypic intensity (0.1) had a musk secretion of 19.4 g, but it should be noted that long-term or increased intensity of stereotypic behaviors may pose health risks. This energy reallocation might occur through reduced investment in non-essential activities (e.g., excessive vigilance) and optimized metabolic pathways [20]. Therefore, in practical management, a tiered strategy should be developed: For individuals with low-intensity stereotypic behaviors (<0.08), appropriate environmental enrichment can be used to maintain stress adaptation, and excessive intervention should be avoided to prevent the disruption of balance. For individuals with high-intensity stereotypic behaviors (≥0.10), priority should be given to more environmental enrichment and welfare enhancement measures [41,42].

This study found no significant differences in personality traits across different age groups of forest musk deer. We divided the individuals into three age groups (1.5, 2.5, and 3.5 years) to capture potential developmental changes during early maturity, as personality traits may stabilize after sexual maturity [21]. The grouping criterion was based on actual age (in years) recorded for each individual, ensuring clear separation of developmental stages. The lack of age-related differences indicates that personality development is largely completed by 1.5 years of age and remains relatively stable thereafter. The presentation of such findings may also be attributed to the limited age range of the sample, which did not cover the complete developmental stages (≥3.5 years), resulting in the age effect not being apparent. Future research should expand the age range to analyze the potential impact of developmental stages on behavior and personality.

This study demonstrated that forest musk deer with higher exploration and activity levels tend to have higher muscone content, indicating better musk quality. Additionally, personality traits of exploration and activity significantly promote the muscone content. This suggests that individuals with high EL and AL are better adapted to the captive environment, resulting in higher musk quantity and quality, which is consistent with previous findings [28]. The high-exploration and high-activity traits of forest musk deer may activate lipid metabolism pathways through frequent exposure to new stimuli, thereby facilitating the muscone synthesis. There is also a general tendency of explorative individuals to travel more and possess large home ranges or its core areas, which is good for their health and welfare [43,44,45]. Therefore, personality traits can serve as predictive indicators for musk secretion in forest musk deer. Thus, in breeding selection, individuals with higher musk quantity and quality can be identified based on their exploratory and activity performance. In the captive environment for forest musk deer, practical measures such as updating feeding devices and environmental enrichment can stimulate exploratory behavior and maintain optimal activity levels [25].

## 5. Conclusions

In summary, the low-intensity stereotypic behaviors observed in this study did not cause damage to the health and musk production of captive musk deer, which may represent an adaptative response to the captive environment. Therefore, one of the key implications of this research is that excessive intervention should be avoided to prevent disrupting this adaptive balance. The intensity of exploratory and active behaviors are important factors in enhancing musk quantity and quality. These two personality traits can be used as predictive indicators for musk secretion in breeding practices. Future research should integrate endocrine indicators, such as cortisol and sex hormones, to elucidate the molecular mechanisms affecting musk secretion. Furthermore, the implementation of a synergistic management model that prioritizes welfare production, based on behaviors and personality traits, could facilitate the development of specialized enclosures, thereby promoting the sustainable breeding of forest musk deer and sustainable musk production. Additionally, future research should include longer-term longitudinal studies to assess cumulative behavioral effects on population dynamics, and comparative studies across different latitudes to understand how environmental factors influence behavioral expression and musk production in captive musk deer.

## Figures and Tables

**Table 1:** Ethogram of stereotypic behaviors in forest musk deer.

Stereotypic Behavior	Description
Feeding on non-food material	The forest musk deer chews or ingests non-nutritive substances such as feces, soil, stones, or fur. This behavior is clearly distinguishable from normal rumination and may include gnawing its own or another individual’s pelage, sometimes producing audible crunching sounds.
Stereotyped licking and scraping	Without obvious environmental stimuli, the forest musk deer suddenly interrupts feeding or resting behavior, frequently shaking its head backwards or rubbing parts of its body with its hind hooves against the wall.
Galloping	Without obvious environmental stimuli, the forest musk deer suddenly interrupts ongoing behavior and runs rapidly within the enclosure, often stopping abruptly or changing direction before switching to unrelated activities such as feeding or lying down.
To-fro-walking	Without obvious stimuli, the forest musk deer moves back and forth within the enclosure at a relatively consistent speed. The starting point and the turning-back point are relatively fixed, and they are not accompanied by other activities, such as sniffing, lasting for more than 30 s.
Platform standing	The forest musk deer stands at a higher position in the enclosure (on cement or wooden bedding) and stares in a certain direction for more than 30 s, sometimes accompanied by slight body movements.
Wall jumping	The forest musk deer jumps vigorously between the wall and the ground of the enclosure, with the take-off and landing points being relatively fixed, and no significant horizontal displacement is observed between multiple jumps.
Stereotypic gazing	Without obvious stimuli, the forest musk deer stands on its hind hooves on the ground of the enclosure, puts its front hooves on vertical surfaces such as cement walls or fences, and gazes at a certain position for more than 30 s, or listens intently in a fixed direction.

**Table 2:** Relationship between the occurrence of stereotypic behaviors and musk secretion.

Age (a)	Whether Stereotypic Behaviors Are Exhibited	*N*	Secretion Amount of Musk (g)	Muscone Content (%)
Total	Yes	11	16.47 ± 6.12	1.75 ± 0.88
Total	No	14	12.14 ± 4.96	1.56 ± 1.02
1.5	Yes	4	10.9 ± 2.9	2.00 ± 0.33
1.5	No	4	8.9 ± 2.2	1.65 ± 0.33
2.5	Yes	4	22.7 ± 2.1	1.98 ± 1.29
2.5	No	6	13.4 ± 4.2	1.51 ± 1.28
3.5	Yes	3	15.6 ± 4.3	1.12 ± 0.40
3.5	No	4	13.6 ± 6.5	1.54 ± 0.94

**Table 3:** The correlation between personality traits and stereotypic behavior.

	EL	AL	SL	RL	SBI
EL	-	0.780 **	−0.317	−0.374	0.296
AL	0.780 **	-	−0.282	−0.256	0.162
SL	−0.317	−0.282	-	−0.192	−0.044
RL	−0.374	−0.256	−0.192	-	−0.399 *
SBI	0.296	0.162	−0.044	−0.399 *	-

Note: Correlation coefficients for EL and SBI are Spearman’s *ρ* (based on non-normal distribution); coefficients for AL, SL, and RL are Pearson’s *r* (based on normal distribution). EL, exploration level; AL, activity level; SL, self-directed level; RL, behavioral redundancy level; SBI, stereotypic behaviors intensity. * *p* < 0.05, ** *p* < 0.01.

**Table 4:** The correlation between personality trait level and musk secretion.

	EL	AL	SL	RL	SAM	MC
EL	-	-	-	-	0.262	0.461 *
AL	-	-	-	-	0.128	0.443 *
SL	-	-	-	-	0.224	−0.288
RL	-	-	-	-	−0.016	−0.104
SAM	0.262	0.128	0.224	−0.016	-	0.184
MC	0.461 *	0.443 *	−0.288	−0.104	0.184	-

Note: Correlation coefficients for EL and SBI are Spearman’s *ρ* (based on non-normal distribution); coefficients for AL, SL, and RL are Pearson’s *r* (based on normal distribution). EL, exploration level; AL, activity level; SL, self-directed level; RL, behavioral redundancy level; SAM, secretion amount of musk; MC, muscone content. * *p* < 0.05.

## Data Availability

The original contributions presented in this study are included in the article. Further inquiries can be directed to the corresponding author.
